# A1 astrocytes contribute to murine depression-like behavior and cognitive dysfunction, which can be alleviated by IL-10 or fluorocitrate treatment

**DOI:** 10.1186/s12974-020-01871-9

**Published:** 2020-07-01

**Authors:** He-Yang Zhang, Yan Wang, Youdi He, Ting Wang, Xiao-Hui Huang, Chang-Ming Zhao, Lei Zhang, Si-Wei Li, Changyong Wang, Yan-Nv Qu, Xiao-Xia Jiang

**Affiliations:** 1 Department of Neural Engineering and Biological Interdisciplinary Studies, Institute of Military Cognition and Brain Sciences, Academy of Military Medical Sciences, 27 Taiping Road, Haidian District, Beijing, 100850 China; 2 Anhui Medical University, Hefei, 230032 Anhui China; 3 Department of Neurology, Beijing Chaoyang Hospital, Capital Medical University, Beijing, 100020 China; 4 College of Agroforestry Engineering and Planning, Tongren University, Tongren, 554300 Guizhou China; 5 Department of Geriatrics, Peking University Shenzhen Hospital, Shenzhen, 518036 Guangzhou China

**Keywords:** A1 astrocytes, IL-10, Neuroinflammation, Behavior deficits, Cognitive dysfunction

## Abstract

**Background:**

Astrocytes are crucial regulators in the central nervous system. Abnormal activation of astrocytes contributes to some behavior deficits. However, mechanisms underlying the effects remain unclear. Here, we studied the activation of A1 astrocytes and their contribution to murine behavior deficits.

**Methods:**

A1 astrocytes were induced by treatment with lipopolysaccharide (LPS) in vitro. The functional phenotype of astrocytes was determined by quantitative RT-PCR, ELISA, and immunohistochemistry. To assess the role of A1 astrocytes in vivo, mice were injected intraperitoneally with LPS. Then, murine behaviors were tested, and the hippocampus and cortex were analyzed by quantitative RT-PCR, ELISA, and immunohistochemistry. The function of IL-10 and fluorocitrate on A1 astrocyte activation was also examined.

**Results:**

Our results show that astrocytes isolated from B6.129S6-Il10^tm1Flv^/J homozygotes (IL-10^tm1/tm1^) were prone to characteristics of A1 reactive astrocytes. Compared with their wild-type counterparts, IL-10^tm1/tm1^ astrocytes exhibited higher expression of glial fibrillary acidic protein (GFAP). Whether or not they were stimulated with LPS, IL-10^tm1/tm1^ astrocytes exhibited enhanced expression of A1-specific transcripts and proinflammatory factors IL-1β, IL-6, and TNFα. In addition, IL-10^tm1/tm1^ astrocytes demonstrated hyperphosphorylation of STAT3. Moreover, astrocytes from IL-10^tm1/tm1^ mice showed attenuated phagocytic ability and were neurotoxic. IL-10^tm1/tm1^ mice demonstrated increased immobility time in the forced swim test and defective learning and memory behavior in the Morris water maze test. Moreover, enhanced neuroinflammation was found in the hippocampus and cortex of IL-10^tm1/tm1^ mice, accompanying with more GFAP-positive astrocytes and severe neuron loss in the hippocampus. Pretreatment IL-10^tm1/tm1^ mice with IL-10 or fluorocitrate decreased the expression of proinflammatory factors and A1-specific transcripts in the hippocampus and cortex, and then alleviated LPS-induced depressive-like behavior.

**Conclusion:**

These results demonstrate that astrocytes isolated from B6.129S6-Il10^tm1Flv^/J homozygotes are prone to A1 phenotype and contribute to the depression-like behavior and memory deficits. Inhibiting A1 astrocyte activation may be an attractive therapeutic strategy in some neurodegenerative diseases.

## Introduction

Astrocytes comprise the most abundant glial cells in the mammalian brain. They are critical regulators of brain development and perform many functions important for normal neuronal survival and growth, synapse formation, and proper propagation of action potentials [1–3].

There is increasing evidence that astrocytes in different brain regions are not identical, and even neighboring astrocytes within the same brain region may have differences [4–6]. Reactive astrocytes, a common pathological response during the repair and scarring of the brain following injury, are often associated with brain metastases in animal models and human patients [7]. Reactive astrocytes have been shown to enhance tumor cell proliferation, survival, invasive capabilities, and resistance to chemotherapy [7]. Recent studies have shown that neuroinflammation and ischemia induced two different types of reactive astrocytes, termed “A1” and “A2” [8–10]. A1 neuroinflammatory reactive astrocytes have been previously shown to be destructive to synapses, suggesting that A1 might have “harmful” functions. By contrast, ischemia-induced A2 reactive astrocytes upregulated many neurotrophic factors, which promote survival and growth of neurons, as well as thrombospondins, which promote synapse repair. This upregulation suggests that A2 might have trophic reparative functions. The finding that different types of reactive astrocytes are induced by different types of injury—and that ischemic injury produces a so-called helpful “A2” reactive astrocyte, whereas an inflammatory insult produces a more harmful “A1” reactive astrocyte—raises many questions [10–12]. A more comprehensive understanding of the heterogeneity of reactive astrocytes still requires a more thorough investigation.

IL-10 is a key cytokine that represses excessive inflammatory responses links with anti-inflammatory and protective functions in the central nervous system (CNS) [13]. In the CNS, IL-10 is mainly produced by astrocytes and microglia and it is upregulated after various insults. Astrocyte-targeted production of IL-10 attenuated the microglial response and culminated in a beneficial effect on neuronal survival [14–16]. Adult neural stem cells (NSCs) engineered to express IL-10 exhibited enhanced capacity to suppress immune response, promote remyelination, and neuronal repair [17]. In addition to ameliorate chronic CNS inflammation, IL-10 modulates progenitor differentiation and regulates neurogenesis [18, 19]. However, elevated IL-10 is not always beneficial. In some Alzheimer’s disease (AD) patient brains, IL-10 signaling pathway was abnormally increased. In APP/PS1 mice, IL-10 deficiency was shown to partially rescue synaptic toxicity and mitigate behavioral impairment [20]. IL-10 overexpression in the brains of APP transgenic mice resulted in increased Aβ accumulation and impaired memory [21]. The complex effect of IL-10 on innate immune activation status in the brain and proteostasis and neurodegenerative diseases indicates controlling the right dose of IL-10 under right condition is critical for brain development and function.

In the present study, we found astrocytes from B6.129S6-Il10^tm1Flv^/J homozygotes (IL-10^tm1/tm1^), which have lower IL-10 expression, were prone to A1 astrocytes, showing as higher levels of A1 transcripts H2-T23, H2-D1, Gbp2, and pro-inflammatory factors, attenuated normal astrocytic protective functions. After treated with lipopolysaccharide (LPS), IL-10^tm1/tm1^ homozygotes showed enhanced neuroinflammation; elevated expression of A1 transcripts H2-T23, H2-D1, and Gbp2; and worse behavioral deficits. Exogenous IL-10 or fluorocitrate (FC) inhibited A1 astrocyte activated and ameliorated LPS-induced behavior deficits.

## Material and methods

### Animals

Groups of 8–12-week-old C57BL/6 mice were obtained from the Laboratory Animal Center of the Academy of Military Medical Sciences of China (Beijing). B6.129S6-Il10^tm1Flv/J^ mice [22] were from Prof. Jianli Wang at Zhejiang University. In all experiments, age- and sex-matched wild-type (WT) littermates were used for controls. Mice were maintained in a pathogen-free barrier facility. All animal experiments were performed according to the Guide for the Care and Use of Laboratory Animals and were approved by the Institute of Military Cognition and Brain Sciences. The institutional Ethics Review Committee for Animal Experimentation approved all experimental protocols.

### Primary cell culture

Primary astrocytes were prepared from newborn C57BL/6 or B6.129S6-Il10^tm1Flv/J^ pups. After the removal of meninges, the cortical tissues were mechanically minced and dissociated with 0.25% trypsin (Gibco). After trypsin inactivation, the tissue suspension was filtered through a 70-mm nylon cell strainer. Cell pellets were harvested and resuspended in Dulbecco’s modified Eagle medium/nutrient mixture F-12 (DMEM/F12) (1:1) (Gibco) supplemented with 10% heat-inactivated FBS, 100 U/ml penicillin, and 100 μg/ml streptomycin and plated on poly-L-lysine pre-coated culture flasks. Following incubation overnight in a humidified incubator at 37 °C and 5% CO_2_, the flasks were vigorously shaken to detach neurons and microglia. Culture medium was replaced every 3 days thereafter until the cell monolayer reached confluence. Primary neurons were obtained by microdissection from brains of neonatal C57BL/6 pups using the same protocol but cultured in neurobasal medium supplemented with 1% glutamate and 2% B27 (Gibco). Primary microglial cells were also dissected from neonatal C57BL/6 pups. After removing the meninges, brains were mechanically minced and dissociated with 0.25% trypsin. The tissue suspension was passed through a 70-mm nylon cell strainer. Cell pellets were harvested and resuspended in DMEM supplemented with 10% heat-inactivated FBS and plated on poly-L-lysine precoated culture flasks. Primary microglial cells were harvested by shaking (200 rpm, 20 min) after overnight in culture.

### Quantitative PCR

Cells were collected in TRIzol (Sigma-Aldrich), and total RNA were prepared by chloroform extraction and isopropanol precipitation according to the manufacturer’s recommendations (Invitrogen). cDNA was used as a template in quantitative PCR with SYBR Green (Toyobo) to determine specific gene expression. Real-time RT-PCR was performed using a SYBR Green PCR mix and conducted with the FTC-3000. Primer sequences were as follows: Aldh1l1 (forward, AGCAGAGGCCATTCACAACT; reverse, GCCACCAGTCCTGAAGTGTT), Aqp4 (forward, CTCCCTTTGCTTTGGACTCA; reverse, CGATGCTGATCTTTCGTGTG; GFAP: forward, GCCACCAGTAACATGCAAGA; reverse, GGCGATAGTCGTTAGCTTCG), Vim (forward, CTGCACGATGAAGAGATCCA; reverse, AGCCACGCTTTCATACTGCT), Aif1 (forward, CCGAGGAGACGTTCAGCTAC; reverse, GACCAGTTGGCCTCTTGTGT). Cx3cr1 (forward, AGCTCACGACTGCCTTCTTC; reverse, GTCCGGTTGTTCATGGAGTT), Snap25 (forward, GAGCAGGTGAGCGGCATCATC; reverse, CGTTGGTTGGCTTCATCAATTCTGG; Syt1: forward, GACTATGACAAGATTGGCAAGAACGAC; reverse, ATGGCATCAACCTCCTCCTCTACC), H2-T23 (forward, TGATCATCCTTGGAGCTGTG; reverse, TTCTGAGGCCAGTCAGAGGT), Serping1 (forward, GCTCTACCACGCCTTCTCAG; reverse, GGATGCTCTCCAAGTTGCTC). H2-D1 (forward, GTTGCTGTTCTGGGTGTCCT; reverse, CCTGGAGCCAGAGCATAGTC), Gbp2 (forward, GGAGGAGCTGTGTGGTGAAT; reverse, TTAGACGTGGCCCATTGACT; Tgm1: forward, CCCTGGATGACAATGGAGTT; reverse, GAATAGCCGGTGCGTAGGTA), Ptx3 (forward, TTCCTGAGGGTGGACTCCTA; reverse, CCGATCCCAGATATTGAAGC), S100a10 (forward, GTGCTCATGGAACGGGAGT; reverse, AAAGCTCTGGAAGCCCACTT). Gpc4 (forward, GGTGACTGTGAAGCCCTGTT; reverse, TCTCTGCCACCATCAGCATA), Gpc6 (forward, AGCATTGCCCTACACCATCT; reverse, AGCCCATCGTTCATGATCTC; Sparcl1: forward, AAACCATCCCAGTGACAAGG; reverse, TCCTCATCCTTCAGGTCCAC), Thbs1 (forward, TGATGACTACGCTGGCTTTG; reverse, TGAGTATCCCTGAGCCCTTG), Thbs2 (forward, CTGGTGCAGACAGCAAACTC; reverse, CAAAGCCAGCGTAGTCATCA). Megf10 (forward, CTGGCGCTGTTCATCATCTA; reverse, CAGGGTTTCTGCGATGGTAT), Mertk (forward, CACGAGAACAACCTTCGTGA; reverse, TCCTCCGGTAAGACACCATC; Gas6: forward, AGGCCACCCTAGAAGTGGAT; reverse, CAGGCCTCCAACATAGGTGT), Axl (forward, GGAAGGTCAGCTCAATCAGG; reverse, CCTTCATGCAGACAGCTTCA), IL-1β (forward, GTGGCTGTGGAGAAGCTGTGG; reverse, CGGAGCCTGTAGTGCAGTTGTC), IFNγ (forward, CAGGCCATCAGCAACAACATAAGC; reverse, AGCTGGTGGACCACTCGGATG; IL-6: forward, ACTTCCATCCAGTTGCCTTCTTGG; reverse, TTAAGCCTCCGACTTGTGAAGTGG), TNFα (forward, GATGGGTTGTACCTTGTCTACT; reverse, CTTTCTCCTGGTATGAGATAGC), iNOS (forward, CAGCTGGGCTGTACAAACCTT; reverse, CATTGGAAGTGAAGCGTTTCG), GAPDH (forward, TCACCATCTTCCAGGAGCGAGAC; reverse, AGACACCAGTAGACTCCACGACATAC).

### Immunofluorescence

Brain processing and immunostaining were performed on free-floating sections. Mice were anesthetized with 2,2,2-tribromoethanol (350 mg/kg, Sigma-Aldrich, T48402) and transcardially perfused with 0.9% saline, and brains were removed and fixed in phosphate-buffered 4% paraformaldehyde for 48 h before cryoprotection with 30% sucrose. Mouse brain sections (30 μm) or fixed cultured cells were washed three times with PBS, and antigen retrieval was performed using citrate buffer (pH 7.0); samples were then permeabilized and blocked in PBS containing 0.5% Triton X-100 and 10% normal goat serum at room temperature for 1 h. Sections were incubated with primary antibodies in blocking buffer overnight at 4 °C. After washing, secondary antibodies were added to the blocking buffer and incubated for 1 h. Samples were then washed and counterstained with 4′,6-diamidino-2-phenylindole (DAPI). Images were acquired under a fluorescence microscope (Nikon AZ-100 multipurpose microscope). Primary antibodies used for immunostaining include GFAP (mouse, 1:500; Abcam, ab10062), IBA1 (Rabbit, 1:300; Abcam, ab178847), and MAP2 (goat, 1:500; Abcam, ab32454). Donkey anti-mouse/rabbit 488/594 secondary antibodies (1:1000) and mounting medium with DAPI were purchased from Invitrogen.

### Western blot

Cells were lysed with lysis buffer, and protein samples were separated on 12% SDS-polyacrylamide gel, and then the proteins were transferred to 0.45 μm polyvinylidene fluoride blotting membranes. The membrane was blocked in 5% non-fat dry milk for 1 h and was then probed overnight at 4 °C with mouse anti-GFAP (1:1000, Abcam, ab10062), rabbit anti-p-STAT3 (1:1000, Cell Signaling Technology, #9167), mouse anti-STAT3 (1:1000, Cell Signaling Technology, #9139), or rabbit anti-GAPDH (1:1000, Cell Signaling Technology, #1228). Blots were incubated with HRP-conjugated secondary antibodies at 1:5000 for 2 h at room temperature and developed using enhanced chemiluminescence substrate (Thermo Fisher, Waltham, MA, USA). Visualization and imaging of blots was performed with a FluorochemQ System (ProteinSimple).

### Neuron survival assays

Astrocytes were treated with or without LPS (100 ng/mL, L-4391, Sigma-Aldrich) for 24 h, and then, supernatants were collected. Neurons were plated at 150,000 cells per well on PDL-coated plastic coverslips in 24-well cell culture plates. Supernatants were added to neurons and viability assessed at indicated time point.

### Aβ phagocytosis

For phagocytosis assays, astrocytes pretreated with or without LPS (100 ng/mL for 24 h) were incubated with aggregated 400 nM Hilyte555-Aβ42 (Anaspec, Fermont, USA). Cells were analyzed at indicated time points following addition of Aβ to the culture.

### LPS administration

Lipopolysaccharide (LPS, L-4391, Sigma-Aldrich) was dissolved in physiological saline. The dose of LPS (0.83 mg/kg) and the time point (24 h) for behavioral tests after LPS intraperitoneal injection (i.p.) were selected based on previous investigations [23].

### Hippocampus and cortex protein extraction

Snap-frozen hippocampus and cortex were homogenized in tissue protein extraction reagent (T-PER, Thermo Fisher Scientific) containing a mixture of protease and phosphatase inhibitors (Thermo Fisher Scientific). Homogenates were centrifuged, and the supernatants were evaluated for the quantification of cytokines.

### ELISA quantification of cytokines

Cytokines were quantified with custom Mouse ProcartaPlex™ Panel according to the manufacturer’s instructions (eBioscience). Data acquisition was performed with a MAGPIX (Luminex). Each sample was measured in duplicate.

### IL-10 and fluorocitrate injection

Mice were anesthetized with 2,2,2-Tribromoethanol (350 mg/kg, Sigma-Aldrich, T48402) intraperitoneal injection, then placed into a stereotaxic frame. A small Hamilton syringe (33-gauge) was used to slowly inject 1 μl of saline or IL-10 (20 ng, Peprotech) or fluorocitrate (FC, 1 nmol, Sigma-Aldrich) per mouse into intracerebroventricular (i.c.v). After injection, the syringe was held in place for 5 min to avoid back-flow of cerebrospinal fluid (CSF). The stereotaxic coordinates were 1.34 mm posterior, 0 mm lateral to the bregma, and 2.3 mm ventral to the bregma.

### Forced swim test (FST)

Mice were individually placed in a glass cylinder (25 cm height, 15 cm diameter) filled with room temperature water (23 °C–25 °C). Mice were allowed to swim inside the cylinder and videotaped for 6 min. The duration of immobility in the last 4 min was counted by an observer blinded to the animal treatments. Immobility was defined as time when animals remained floating or motionless with only movements necessary for keeping balance in the water.

### Morris water maze test (MWM)

The water maze used in this study comprised a circular tank 120 cm in diameter with a platform filled with tap water at a temperature of 22 ± 2 °C. Reference cues with different colors and shapes were posted along the walls surrounding the tank. Within the tank was a fixed platform (diameter, 10 cm) located in a target quadrant. A camera was mounted above the maze to record the swimming traces in the water maze. During testing, the platform was submerged, and 1–2 cm below the water surface, mice were placed into the maze at one of four points (N, S, E, W) facing the wall of the tank. Mice were provided 60 s to search for the platform. If a mouse failed to find the platform, it was guided to the platform and maintained on the platform for 10 s. Four trials per day were conducted with an intermission of 1 h minimum between trials. Between the trails, mice were gently padded dry and warmed on a heating pad. The escape latency, or the time it took for the mouse to reach the platform, was scored for each trial and averaged per testing day. On day 6, the platform was removed and a probe test was performed. The percentage of time spent in each of the four quadrants and the number of platform area crossings, mean speed, and total distance were recorded.

### Statistical analysis

All data were analyzed with Prism 5.0 software (GraphPad Software, San Diego, CA, USA) and are presented as means ± standard deviations (SDs). Statistical significance was assessed by unpaired two-tailed Student’s *t* tests. For multiple comparisons, two-way ANOVA was performed, followed by Bonferroni multiple comparison. (*p < 0.05; **p < 0.01).

## Results

### A1-specific transcripts were induced in astrocytes with inflammatory factors

**Fig. 1:**
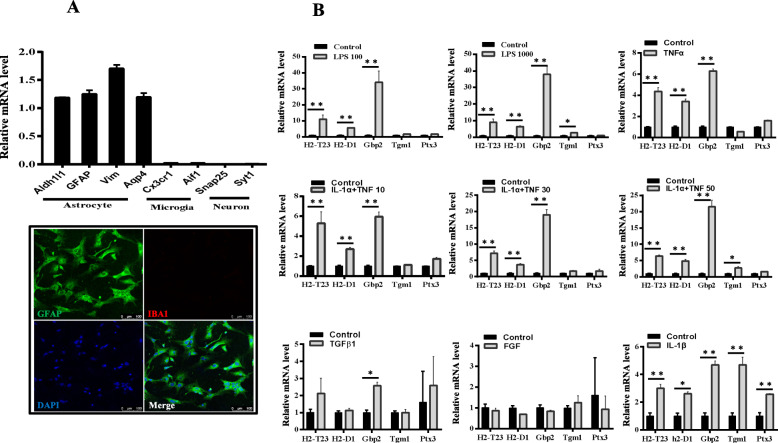
A1-specific transcripts were induced in astrocytes. **a** Representative graph showing the relative mRNA levels of the indicated factors from astrocytes isolated from neonatal mice (top). Immunofluorescent staining for GFAP and IBA1 on cultured astrocytes (bottom). **b** Representative graph showing the relative mRNA levels of the indicated factors. Astrocytes were either untreated or treated with indicated doses of LPS (100 ng/mL, 1000 ng/mL), TNFα (30 ng/mL), IL-1α (10 ng/mL) plus TNFα (10 ng/mL, 30 ng/mL, 50 ng/mL), TGFβ (3 ng/mL), FGF (20 ng/mL), or IL-1β (30 ng/mL) for 24 h and then collected for quantitative RT-PCR. Data shown are means ± SD of two independent experiments. *p < 0.05 and **p < 0.01

Previous studies have shown that A1 astrocytes can be induced by multiple inflammatory factors [8]. To confirm the activation state of astrocytes with different stimulators, we first isolated astrocytes from neonatal mice and used quantitative RT-PCR to determine lineage-specific gene expression. As expected, astrocyte-specific markers Aldh1l1, glial fibrillary acid protein (GFAP), Vim, and Aqp4 were highly expressed, while microglia marker Cx3cr1, Aif1 and neuron marker Snap25, Syt1 were almost undetectable (Fig. 1a, top). GFAP and microglial marker, ionized calcium-binding adaptor molecule 1 (IBA1) revealed that no microglial cells were detected in astrocytic cultures, excluding a potential contribution of microglia in the culture system (Fig. 1a, bottom). Astrocytes can be activated by various stimulators. We treated the isolated astrocytes with different doses of LPS, TNFα, IL-1α plus TNFα, TGF-β1, FGF, and IL-1β, respectively, for 24 h. As shown in Fig. 1b, A1-specific transcripts H2-T23, H2-D1, and Gbp2 were significantly increased under treatment with LPS, TNFα, IL-1α plus TNFα, and IL-1β. However, A2-specific transcripts only increased significantly under IL-1β treatment. Since LPS at 100 ng/ml can induce high expression of A1-specific transcripts, the stimulation condition was chosen for the following studies.

### Astrocytes from IL-10^tm1/tm1^ mice were prone to A1 phenotype

**Fig. 2:**
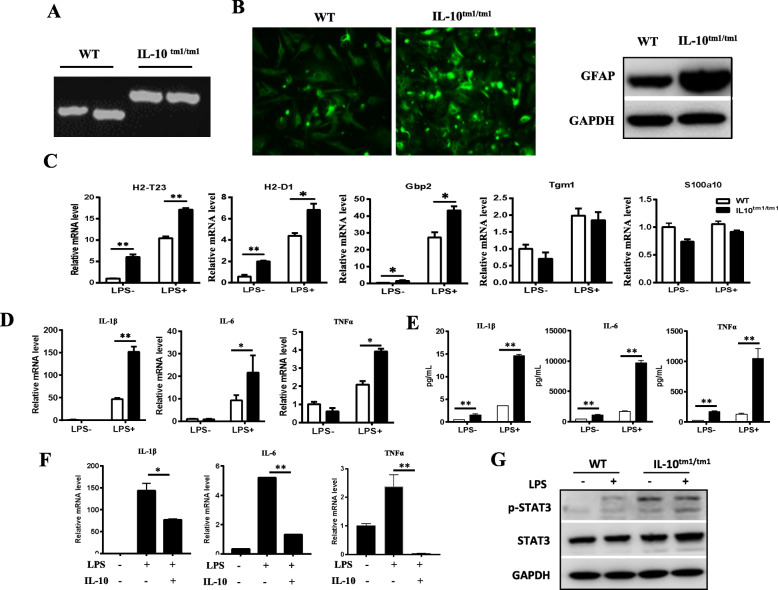
Astrocytes from IL-10^tm1/tm1^ mice were prone to the A1 phenotype. **a** Genotyping of WT and homozygous IL-10^tm1/tm1^ mice. **b** Representative immunofluorescent staining (right) and representative western blots for GFAP in astrocytes from WT and IL-10^tm1/tm1^ mice. Representative graph showing relative mRNA levels of indicated transcripts (**c**) and pro-inflammatory factors (**d**) in primary cultured astrocytes isolated from WT and IL-10^tm1/tm1^ mice stimulated with or without 100 ng/mL LPS for 24 h. **e** Protein levels of the indicated cytokines in the supernatants of primary cultured astrocytes isolated from WT and IL-10^tm1/tm1^ mice which stimulated with or without 100 ng/mL LPS for 24 h. **f** Representative graph showing relative mRNA levels of indicated pro-inflammatory cytokines in astrocytes isolated from IL-10^tm1/tm1^ mice treated with the indicated factors. Data shown are means ± SD of two independent experiments. **g** Representative western blots showing the expression of indicated proteins in astrocytes from WT and IL-10^tm1/tm1^ mice treated with or without LPS. *p < 0.05 and **p < 0.01

IL-10 is a kind of anti-inflammatory factor and has a complex effect on innate immune activation status in the brain. To determine the role of IL-10 in astrocytes, B6.129S6-Il10^tm1Flv^/J homozygotes (IL-10^tm1/tm1^) [22], which have reduced IL-10 expression, were used in this study. PCR data confirmed the genotype of IL-10^tm1/tm1^ mice (Fig. 2a). Astrocytes were isolated from neonatal WT and IL-10^tm1/tm1^ mice and then assessed for GFAP expression. Both immunofluorescence staining (Fig. 2b, left) and immunoblot data (Fig. 2b, right) demonstrated a higher level of GFAP in IL-10^tm1/tm1^ astrocytes than in WT astrocytes. In addition, IL-10^tm1/tm1^ astrocytes showed high expression of A1 transcripts H2-T23, H2-D1, and Gbp2 under normal culture conditions. As expected, when stimulated with LPS, A1 transcripts H2-T23, H2-D1, and Gbp2 were significantly induced. However, higher expression was detected in IL-10^tm1/tm1^ astrocytes than in their WT counterparts, while A2 transcripts Tgm1 and S100a10 were comparable in WT and IL-10^tm1/tm1^ astrocytes (Fig. 2c). Next, we examined the expression of pro-inflammatory factors in IL-10^tm1/tm1^ and WT astrocytes. Quantitative RT-PCR and ELISA analysis revealed that when stimulated with LPS, the expression of pro-inflammatory factors, including IL-1β, IL-6, and TNFα, was increased. Compared with their WT counterparts, IL-10^tm1/tm1^ astrocytes demonstrated significantly enhanced expression of IL-1β, IL-6, and TNFα (Fig. 2d, e). The LPS-induced expression of pro-inflammatory factors in IL-10^tm1/tm1^ astrocytes was reduced by exogenous administration of IL-10 (Fig. 2f). In the CNS, STAT3 is a good candidate to be an activator of certain aspects of astrogliosis. As shown in Fig. 2g, coincident with higher expression of GFAP (Fig. 2b), phosphorylation of STAT3 was also enhanced in IL-10^tm1/tm1^ astrocytes, even stronger than that in WT astrocytes with LPS treatment.

### Astrocytes from IL-10^tm1/tm1^ mice showed attenuated normal astrocytic functions

**Fig. 3:**
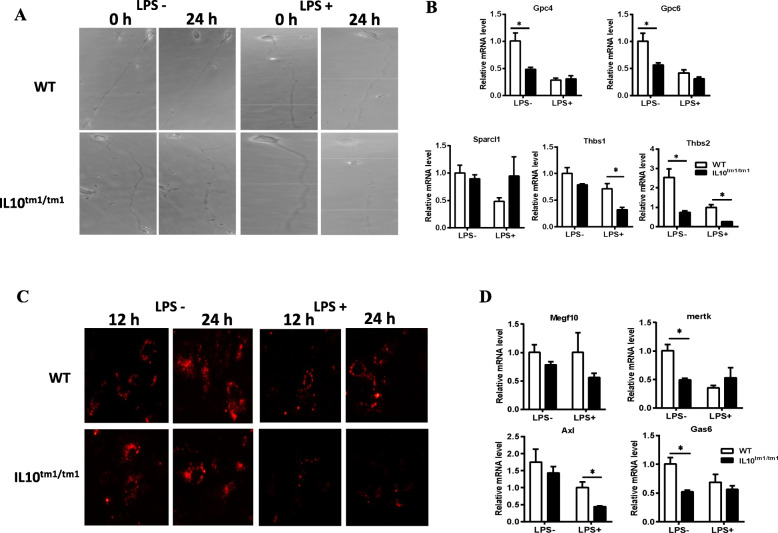
Astrocytes from IL-10^tm1/tm1^ mice showed attenuated normal astrocytic functions. **a** Representative phase images of neurons grown in culture medium supplied with supernatant from WT and IL-10^tm1/tm1^ astrocytes treated with or without LPS. **b** Representative graph showing relative mRNA levels of indicated factors in primary cultured astrocytes isolated from WT and IL-10^tm1/tm1^ mice which stimulated with or without LPS. **c** Representative fluorescent images of cultured astrocytes engulfing Aβ42. **d** Representative graph showing relative mRNA levels of indicated astrocyte-specific phagocytic receptors and bridging molecules. Data shown are means ± SD of two independent experiments. *p < 0.05 and **p < 0.01

Astrocytes are able to support neuron survival and induce the formation of functional synapses by secreting glypicans (Gpc4 and Gpc6), Sparcl1, and thrombospondins (Thbs1 and Thbs2). Next, we investigated the function of IL-10^tm1/tm1^ astrocytes on neurons. WT and IL-10^tm1/tm1^ astrocytes were stimulated with or without LPS for 24 h, and then, their supernatant was collected. Purified neurons cultured from neonatal mice were treated with supernatant from WT and IL-10^tm1/tm1^ astrocytes. In contrast to support neuron survival, neurons treated with IL-10^tm1/tm1^ astrocyte supernatant lost synaptic integrity (Fig. 3a). Correspondingly, LPS-treated astrocytes showed significantly decreased expression of Gpc4, Gpc6, and Thbs2 mRNA (Fig. 3b). Without LPS stimulation, IL-10^tm1/tm1^ astrocytes demonstrated lower expression of Gpc4, Gpc6, and Thbs2 than WT astrocytes but were comparable to WT astrocytes under LPS treatment. After treatment with LPS, IL-10^tm1/tm1^ astrocytes showed even lower expression of Gpc4, Gpc6, Thbs1, and Thbs2 (Fig. 3b). To examine the phagocytic ability of WT and IL-10^tm1/tm1^ astrocytes, engulfment of Aβ42 was measured. Astrocytes can robustly phagocytose Aβ42, but upon LPS stimulation, the ability significantly decreased (Fig. 3c). Compared with WT astrocytes, IL-10^tm1/tm1^ astrocytes engulfed less Aβ42 whether or not they were treated with LPS. The phagocytic capacity corresponds with the expression of astrocyte-specific phagocytic receptors and bridging molecules. Decreased Mertk and Axl mRNA expression was found in IL-10^tm1/tm1^ astrocytes, especially after LPS stimulation (Fig. 3d).

### IL-10^tm1/tm1^ mice showed higher expression of A1-specific transcripts, enhanced neuroinflammation, and more neuron death

**Fig. 4:**
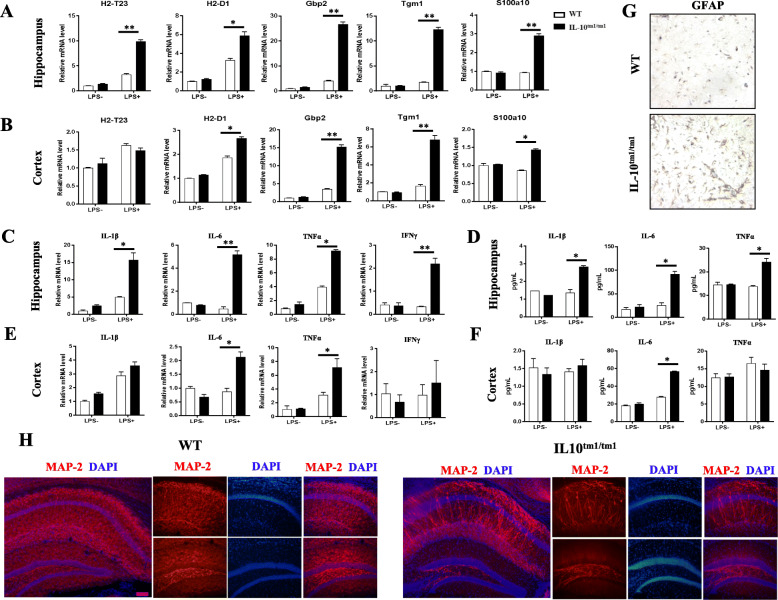
Higher expression of A1-specific transcripts and pro-inflammatory factors and severe neuron death in IL-10^tm1/tm1^ mice. Representative graph showing relative mRNA levels of indicated transcripts (**a**, **b**) and pro-inflammatory factors (**c**, **e**) in the hippocampus (**a**, **c**) and cortex (**b**, **e**) from WT and IL-10^tm1/tm1^ mice with i.p. injection with or without LPS. Protein levels of the indicated cytokines in the hippocampus (**d**) and cortex (**f**) from WT and IL-10^tm1/tm1^ mice subject to i.p. injection with or without LPS. **g** Representative immunohistochemistry staining for GFAP in WT and IL-10^tm1/tm1^ mice. **f** Representative immunofluorescent staining for MAP2 in the hippocampus of WT and IL-10^tm1/tm1^ mice. *p < 0.05 and **p < 0.01

To examine whether lower IL-10 expression will affect the A1 phenotype and neuroinflammation in vivo and then contribute to neural death, the hippocampus and cortex were collected 24 h after intraperitoneal (i.p.) administration of LPS for quantitative RT-PCR analysis. As shown in Fig. 4a, b, A1-specific transcripts H2-T23, H2-D1, and Gbp2 in the hippocampus and H2-D1 and Gbp2 in the cortex were significantly induced in IL-10^tm1/tm1^ mice. Accordingly, quantitative RT-PCR (Fig. 4c) and ELISA (Fig. 4d) analysis revealed that the expression of pro-inflammatory factors IL-1β, IL-6, and TNFα in the hippocampus was significantly higher than those from WT mice. In the cortex, higher levels of IL-6 and TNFα mRNA (Fig. 4e) and IL-6 protein (Fig. 4f) were found in IL-10^tm1/tm1^ mice. In addition, immunofluorescent staining data demonstrated that more GFAP-positive cells were present in IL-10^tm1/tm1^ mice than in WT mice, which indicated the activation of astrocytes (Fig. 4g). A1 astrocytes and neuroinflammation will lead to neuron death. MAP2 immunostaining data revealed that compared with their WT counterparts, loss of MAP2-positive cells was observed in IL-10^tm1/tm1^ mice (Fig. 4h).

### IL-10^tm1/tm1^ mice exhibited depressive-like behavior and learning and memory deficits

**Fig. 5:**
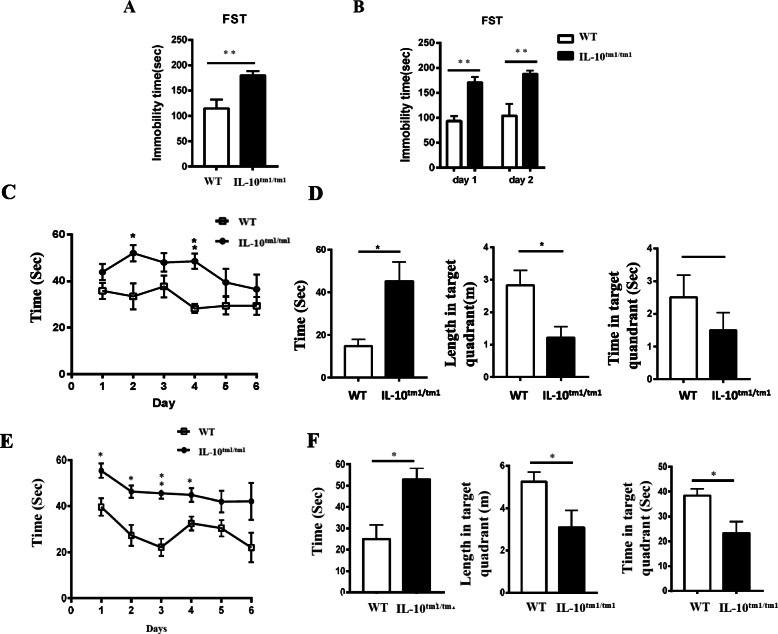
IL-10^tm1/tm1^ mice exhibited depressive-like behavior and learning and memory deficits. **a** Representative graph showing the duration of immobility during the FST without LPS treatment. **b** Representative graph showing the duration of immobility during the FST recorded 24 h and 48 h following administration of LPS. MWMT performed over 6 days, including 5 days training and the 1-day test. The graphic shows the time (seconds) needed for each group of mice without (**c**) or with (**e**) LPS administration to localize a submerged platform in the swimming area. Representative graphs show escape latency (left), path length (middle), and time (right) spending in the target quadrant on the test day for each group of mice without (**d**) or with (**f**) LPS administration. *p < 0.05 and **p < 0.01

Studies have shown that A1 astrocytes are harmful and toxic to neurons and then affect the behaviors [5]. We then assessed potential depressive-like behaviors in IL-10^tm1/tm1^ mice using the forced swim test (FST), which is a reliable behavioral assay for detecting depressant potential. Surprisingly, compared with their WT counterparts, IL-10^tm1/tm1^ mice showed a significant increase in immobility duration during the FST (Fig. 5a). LPS treatment can induce A1 astrocytes. We investigated the effect of LPS on the behaviors of both WT and IL-10^tm1/tm1^ mice. After i.p. injection with LPS, there were more significant differences between the two groups, with increased time of immobility in IL-10^tm1/tm1^ mice during FST, indicating severe depressive-like behavior (Fig. 5b). We then examined the spatial learning and memory of these mice through the Morris water maze test (MWMT) which reflects the functions of brain regions involved in learning and memory such as the hippocampus. Compared with WT mice, IL-10^tm1/tm1^ mice spent a significantly longer time exploring the quadrant that previously contained the platform and had a less number of crossings over the platform location (Fig. 5c, d, e, f). Together, these tests indicated that IL-10^tm1/tm1^ mice exhibited depressive-like behavior and learning and memory deficits.

### IL-10 or FC treatment inhibited A1 astrocyte activation and ameliorated LPS-induced behavior deficits

**Fig. 6:**
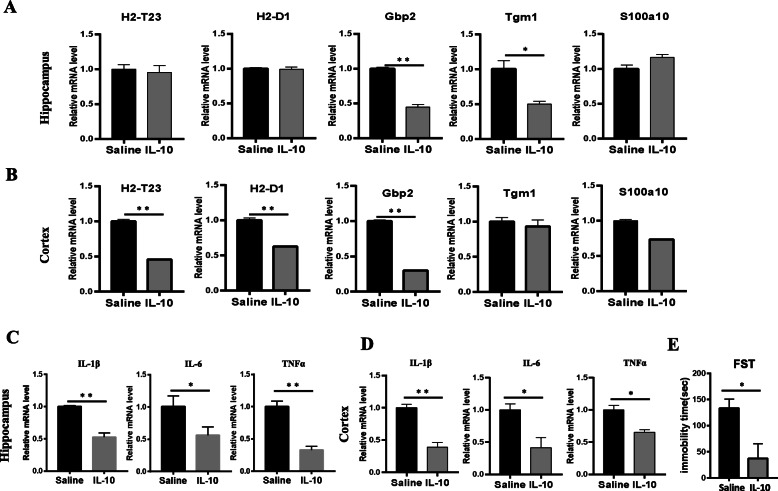
IL-10 treatment inhibited A1 astrocyte activation and ameliorated LPS-induced behavior deficits. Mice were injected i.c.v with normal saline or IL-10 (20 ng/μl per mouse), and 6 h later, mice also received either an i.p. injection of saline or LPS (0.83 mg/kg). FST was performed 24 h following administration of LPS. Immediately following behavioral tests, mice were killed and perfused with ice-cold phosphate-buffered saline, and the hippocampus (**a**, **c**) and cortex (**b**, **d**) were rapidly collected and measured by real-time PCR for transcription factors (**a**, **b**) and inflammatory factors (**c**, **d**). The duration of immobility during the FST (**e**) was recorded 24 h following administration of LPS. Data were expressed as the mean ± SD. *p < 0.05 and **p < 0.01

**Fig. 7:**
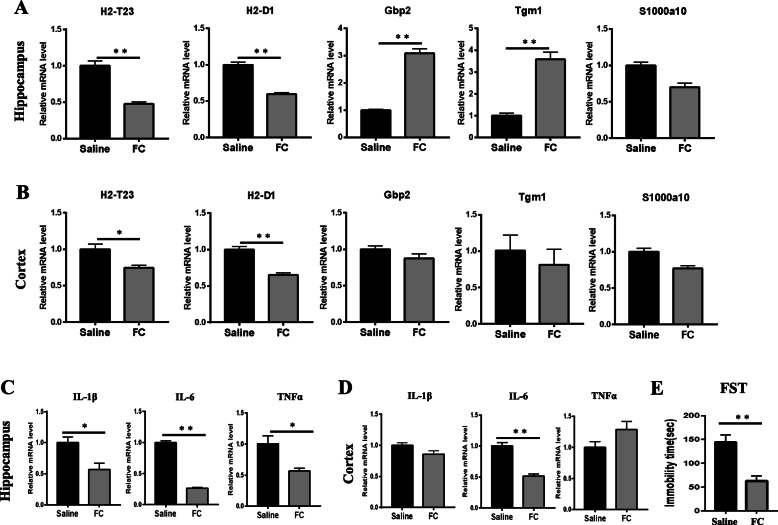
FC treatment inhibited A1 astrocyte activation and ameliorated LPS-induced behavior deficits Mice were injected i.c.v with normal saline or FC solution (1 nmol per mouse), and 6 h later, mice also received either an i.p. injection of saline or LPS (0.83 mg/kg). The FST was performed 24 h following administration of LPS. Immediately following behavioral tests, mice were killed and perfused with ice-cold phosphate-buffered saline, and the hippocampus (**a**, **c**) and cortex (**b**, **d**) were rapidly collected and measured by real-time PCR for transcription factors (**a**, **b**) and inflammatory factors (**c**, **d**). The duration of immobility during the FST (**e**) was recorded 24 h following administration of LPS. Data were expressed as the mean ± SD. *p < 0.05 and **p < 0.01

Previous studies have shown that FC can inhibit the activation of astrocytes and then ameliorates LPS-induced depressive-like behavior [24, 25]. To confirm the effect of A1 astrocyte activation and then on mouse behavior deficits, we analyzed the expression of A1 astrocyte-specific transcripts and pro-inflammatory cytokines in brain samples with IL-10 or FC pretreatment. IL-10^tm1/tm1^ mice were administered with IL-10 (20 ng, i.c.v), FC (1 nmol, i.c.v), or physiological saline for 6 h followed by a single i.p. injection of LPS (0.83 mg/kg). At 24 h after LPS injection, mice were submitted to tests for depressive-like behaviors and then were euthanized for collection of hippocampus and cortex samples. Quantitative RT-PCR data showed that after IL-10 pretreatment, the expression of A1-specific transcripts Gbp2 in the hippocampus (Fig. 6a) and H2-T23, H2-D1, and Gbp2 in the cortex (Fig. 6b) were significantly decreased. Accordingly, attenuated expression of IL-1β, IL-6, and TNFα was found both in the hippocampus (Fig. 6c) and cortex (Fig. 6d) from IL-10-pretreated IL-10^tm1/tm1^ mice. Compared with saline-pretreated mice, IL-10 pretreatment significantly decreased the duration of immobility in the FST (Fig. 6e). Previous studies have shown that FC could inhibit astrocyte activation. Similarly, after FC pretreatment, the expression of A1-specific transcripts H2-T23 and H2-D1 in the hippocampus (Fig. 7a) and H2-T23 and H2-D1 in the cortex (Fig. 7b) were significantly decreased. Attenuated expression of IL-1β, IL-6, and TNFα was also found both in the hippocampus (Fig. 7c), and lower IL-6 was observed in the cortex (Fig. 7d) from IL-10-pretreated IL-10^tm1/tm1^ mice. Compared with saline-pretreated mice, FC pretreatment also significantly decreased the duration of immobility in the FST (Fig. 7e).

## Discussion

In this study, we found that astrocytes from IL-10^tm1/tm1^ mice, which have reduced IL-10 expression, were prone to A1 phenotype. After LPS injection, more A1 astrocytes were present in the brain tissues of IL-10^tm1/tm1^ mice, which might contribute to their severe depressive-like behavior and learning and memory deficits. In addition, our data showed that IL-10 or FC pretreatment partially reduced the activation of A1 astrocytes and then ameliorated their behavior dysfunctions.

Astrocytes are abundant cells in the CNS and can be activated by many different types of molecules [4, 5, 10, 11, 26–29]. Here, we treated astrocytes isolated from neonatal mice with different cytokines and found that LPS, TNFα, or IL-1α plus TNFα can significantly induce the expression of A1 transcripts H2-T23, H2-D1, and Gbp2 but not A2 transcripts Tgm1 and Ptx3, which is similar to previous reports [8]. With LPS stimulation, astrocytes showed enhanced production of proinflammatory factors; decreased expression of Gpc4, Gpc6, Thbs1, and Thbs2; and reduced phagocytic ability. These indicated that the astrocytes in response to LPS showed an A1 characteristic [8, 9, 24, 30]. B6.129S6-Il10^tm1Flv^/J mice are generally used to detect and monitor cells committed to IL-10 production [22]. The homozygotes have reduced IL-10 expression. We found that astrocytes from B6.129S6-Il10^tm1Flv^/J homozygotes (IL-10^tm1/tm1^) were prone to A1 astrocytes. Under normal culture condition, IL-10^tm1/tm1^ astrocytes demonstrated high expression of A1 transcripts. After treatment with LPS, both WT and IL-10^tm1/tm1^ astrocytes exhibited increased expression of A1 transcripts and proinflammatory factors, but the levels in IL-10^tm1/tm1^ astrocytes were much higher. In addition, compared to their WT counterparts, IL-10^tm1/tm1^ astrocytes were more toxic to neurons and less phagocytic, showing the characteristics of A1 phenotypes. What is more, compared with their counterparts, higher expression of A1 transcripts and proinflammatory factors was found in the hippocampus and cortex from IL-10^tm1/tm1^ mice. Liddelow et al. [8] have reported that reactive A1 astrocyte formation is a fundamental pathological response of many neurodegenerative diseases. A1 reactive astrocytes accumulated in aging brain and contribute to cognitive decline and a loss of memory. In addition to more severe depression-like behavior, IL-10^tm1/tm1^ mice also exhibited more severe cognitive and memory deficits after being injected with the neuroinflammation inducer LPS. The behavior tests further confirmed the presence of A1 active astrocytes which secreted inflammatory factors IL-1β, IL-6, IFNγ, and TNFα and some unknown toxic factors inducing neurodegeneration, though we could not exclude the potential contribution of microglia cells in the brain.

Immune dysfunction is commonly associated with several neurological and mental disorders [31–36]. The innate immune system plays a central role in the CNS inflammation that drives neurological disability in progressive forms of multiple sclerosis, for which there are no effective treatments. It has been reported that cytokine imbalance is involved in the progression of many CNS diseases such as neuropsychiatric disorders and neurodegenerative disorders [13, 34, 37]. Enhanced IL-1β production in astrocytes is associated with the pathogenesis of major depressive disorder [38]. IFN-γ-deficient mice exhibited aberrant hyper-connectivity in frontal-cortical/insular regions though did not show anxiety or motor deficits [39]. SCID mice lacked social preference for a mouse over an object, but SCID mice did not show anxiety. IL-4-deficient mice did not demonstrate social deficits; in fact, they spent more time investigating a novel mouse than a novel object compared with WT mice. Further, IL-6 knockout mice show no difference in depressive-like behavior as measured by tail suspension or forced swim tests [40]. Altered IL-6 expression alone cannot account for depressive symptom, but clinical and animal studies showed that increased IL-6 and decreased IL-10 will lead to depression [31, 41, 42]. IL-10-deficient female mice but not male mice displayed increased depressive-like behavior [43]. However, in our study, we found IL-10^tm1/tm1^ male mice with IL-10 low expression exhibited increased immobility time in the forced swim test and defective learning and memory behaviors in the Morris water maze test. The activation of A1 astrocytes and the high expression of inflammatory factors IL-1β, IL-6, IFNγ, and TNFα were mostly associated with the behavior deficits. Similar to some reports, mice pretreated with IL-10 presented a decreased depressive-like behavior [41, 43].

IL-10 is a cytokine classically linked with anti-inflammatory and protective functions in the CNS in different neurodegenerative and neuroinflammatory conditions [15]. In the CNS, IL-10 is mainly produced by astrocytes and microglia and it is upregulated after various insults, such as experimental autoimmune encephalomyelitis, middle cerebral artery occlusion, excitotoxicity, and traumatic brain injury. Astrocyte-targeted production of IL-10 impacted the microglial response and lymphocyte recruitment and culminated in a beneficial effect on neuronal survival [16]. However, elevated IL-10 is not always beneficial. In some AD patient brains, IL-10 signaling pathway was abnormally increased. In APP/PS1 mice, IL-10 deficiency was shown to partially rescue synaptic toxicity and mitigate behavioral impairment [20]. IL-10 overexpression in the brains of APP transgenic mice resulted in increased Aβ accumulation and impaired memory [21]. The complex effect of IL-10 on innate immune activation status in the brain, proteostasis, and neurodegenerative diseases indicates controlling the right dose of IL-10 in the right condition is critical for brain development and function.

## Conclusion

In conclusion, our data demonstrate that IL-10, an anti-inflammatory cytokine, is an important molecule in the modulation of A1 astrocyte activation, depressive-like behavior, and learning and memory dysfunction. Furthermore, our data indicate that reducing the activation of A1 astrocytes may be an attractive therapy for some neuropsychiatric disorders or cognitive and memory deficits.

## Data Availability

The datasets used and/or analyzed during the current study are available from the corresponding author upon reasonable request.

## Abbreviations

GFAP: Glial fibrillary acidic protein
LPS: Lipopolysaccharide
CNS: Central nervous system
NSCs: Neural stem cells
AD: Alzheimer’s disease
FC: Fluorocitrate
DAPI: 4′,6-Diamidino-2-phenylindole
CSF: Cerebrospinal fluid
IBA1: Ionized calcium-binding adaptor molecule 1
FST: Forced swim test
MWMT: Morris water maze test
